# Improved Performance of Compartments in Detecting the Activity of Axial Spondyloarthritis Based on IVIM DWI with Optimized Threshold *b* Value

**DOI:** 10.1155/2022/2276102

**Published:** 2022-01-10

**Authors:** Qiang Ye, Zhuoyao Xie, Chang Guo, Xing Lu, Kai Zheng, Yinghua Zhao

**Affiliations:** Department of Radiology, The Third Affiliated Hospital of Southern Medical University (Academy of Orthopedics, Guangdong Province), Guangzhou, Guangdong 510630, China

## Abstract

**Purpose:**

To explore the diagnostic performance of the optimized threshold *b* values on IVIM to detect the activity in axial spondyloarthritis (axSpA) patients.

**Method:**

40 axSpA patients in the active group, 144 axSpA patients in the inactive group, and 20 healthy volunteers were used to evaluate the tissue diffusion coefficient (*D*_slow_), perfusion fraction (*f*), and pseudodiffusion coefficient (*D*_fast_) with *b* thresholds of 10, 20, and 30 s/mm^2^. The Kruskal-Wallis test and one way ANOVA test was used to compare the different activity among the three groups in axSpA patients, and receiver operating characteristic (ROC) curve analysis was applied to evaluate the performance for *D*_slow_, *f*, and *D*_fast_ to detect the activity in axSpA patients, respectively.

**Results:**

*D*_slow_ demonstrated a statistical difference between two groups (*P* < 0.05) with all threshold *b* values. With the threshold b value of 30 s/mm^2^, *f* could discriminate the active from control groups (*P* < 0.05). *D*_slow_ had similar performance between the active and the inactive groups with threshold *b* values of 10, 20, and 30 s/mm^2^ (AUC: 0.877, 0.882, and 0.881, respectively, all *P* < 0.017). Using the optimized threshold *b* value of 30 s/mm^2^, *f* showed the best performance to separate the active from the inactive and the control groups with AUC of 0.613 and 0.738 (both *P* < 0.017) among all threshold *b* values.

**Conclusion:**

*D*_slow_ and *f* exhibited increased diagnostic performance using the optimized threshold *b* value of 30 s/mm^2^ compared with 10 and 20 s/mm^2^, whereas *D*_fast_ did not.

## 1. Introduction

Axial spondyloarthritis (axSpA) is a chronic inflammatory disease involving predominantly the sacroiliac joints (SIJ), the axial skeleton, and entheses [1], ultimately leading to significant disability and loss of social function [2]. According to data from multiple countries, the incidence of axSpA is 0.4-14 per 100,000 person-years [3]. However, no available agents have demonstrated a disease-modifying effect in axSpA [4]. Hence, an increasing number of treatment trials in axSpA are being conducted, heralding the potential availability of more effective treatments in the future. To make it possible for the comparison between trials of different agents, a noninvasive and quantitative method for evaluating the disease activity of patients with axSpA is highly desirable.

Magnetic resonance imaging (MRI) is an imaging modality of choice for assessing disease activity in axSpA [5]. Furthermore, disease activity in axial spondyloarthritis (axSpA) has been longitudinally associated with SIJ inflammation on MRI [6]. Le Bihan et al. [7] reported that the intravoxel incoherent motion (IVIM) diffusion-weighted imaging (DWI), respectively, measured the pure diffusion coefficient (*D*_slow_), perfusion-related incoherent microcirculation coefficient (*D*_fast_), and perfusion fraction (*f*). The diffusion of molecules is restricted in sacroiliitis since increased protein-rich fluids and inflammatory cells accumulate within the lesion in axSpA. The perfusion is also known to be raised because of the higher amount of blood or serum of capillary transported to the marrow cavity within the lesion in axSpA [8]. So, IVIM DWI was mainly proposed to functionally and qualitatively diagnose the activity of axSpA [9]. But there has been much controversy surrounding the roles of *D*_fast_ and *f* for separating the active from nonactive patients with axSpA [10–12].

In previous studies, quantitative metrics of IVIM were turned out to be not very accurate, partially due to the limited sampling, low signal-to-noise ratio (SNR) for fast data acquisition, diffusion gradient polarity, and so on [13, 14]. First of all, some studies confirmed that segmented-unconstrained analysis should be preferred in the cases of a limited number of *b* values and finite SNRs. Tissue parameters derived from IVIM analysis depend on the threshold *b* value. With the analysis of data sampled from healthy individuals, the optimal threshold *b* value for liver IVIM analysis has been reported [15]. It had been demonstrated that the optimal threshold *b* value of 60 s/mm^2^ could potentially provide better detection for liver fibrosis than threshold *b* values of 40, 80, 100, 150, and 200 s/mm^2^ [16]. However, the optimal threshold *b* value for sacroiliitis IVIM analysis is still unclear. Therefore, in the current study, we explored how the selection of threshold *b* value impacts *f*, *D*_slow_, and *D*_fast_ values and how threshold *b* value impacts IVIM technique's performance to detect the activity of axSpA patients.

## 2. Materials and Methods

### 2.1. Study Population

This prospective study was approved by our Institute Ethics Committee, and written informed consent was obtained from all participants (IRB number AN16327-001). According to the Assessment of Spondyloarthritis International Society criteria for axSpA [17], all patients diagnosed with axSpA were enrolled during the period from August 2017 to August 2019. The patients underwent erythrocyte sedimentation rate (ESR), C-reactive protein (CRP), and Spondyloarthritis Disease Activity Score (ASDAS). Within a week after the laboratory tests and clinical assessments, an MRI examination of the sacroiliac joints was performed using 3.0 T scanner. The exclusion criteria were defined as follows: (1) participants with contraindications cannot be scanned by MRI scanner; (2) patients with axial axSpA had peripheral joint involvement, such as hip, shoulder, and knee joints; (3) patients without IVIM DWI examination; and (4) MR images with low signal-to-noise ratio (SNR). Based on ASDAS and CRP [18], all patients with AS were classified as the active and inactive groups according to the following: the active group, ASDAS-CRP ≥ 1.3, and the inactive group, ASDAS-CRP < 1.3 [19]. Meanwhile, 20 male healthy volunteers (16 males, 4 females; mean age 26.20 ± 5.71 years, range 21-47 years; mean weight 72.85 ± 20.40 kg, range 48–130 kg; mean height 173.8 ± 7.15 cm, range 168–190 cm) were enrolled as the control group. The volunteers were included with the criteria: (1) without a history of low back pain or trauma and (2) without a metallic foreign body.

### 2.2. MR Imaging Techniques

A 3.0Tesla Achieva MR imaging system (Philips Healthcare, Best, Netherlands) equipped with 32 channels body phased array coil was performed to enable the acquisition of high-resolution medical images. In this section, five standard MRI sequences were obtained: (a) Dixon fat-water separation coronal TSE T1WI (TSE T1WI mDixon), (b) Dixon fat-water separation coronal TSE T2WI (TSE T2WI mDixon), (c) axial T1-weighted turbine spin echo (T1-TSE), (d) axial T2-weighted spectral attenuated inversion recovery (T2W-SPAIR), and (e) axial IVIM DWI (Table 1). IVIM DWI was based on a single-shot DW spin-echo echo-planar imaging (EPI) with scanning time of 182 seconds.

### 2.3. Image Postprocessing and Measurement Analysis

A professional image postprocessing workstation with high computing power (PRIDE DWI Tool, IDL Virtual Machine Version 6.3, Philips Healthcare, Japan) was used to calculate complex image algorithm problems. The MRI medical images were imputed to the image postprocessing workstation for further processing.

Two radiologists with 10 and 3 years of experience in musculoskeletal MRI interpretation were involved for the recognition of lesions and delineation of ROIs independently, and then all parameters were acquired from IVIM DWI. Patients' clinical information were blinded to either radiologist. Intra- and interobserver agreement for 40 random patients (20 in the active group and 20 in the inactive group) were evaluated using the intraclass correlation coefficient (ICC) by the two radiologists. ICCs ≥ 0.75 were considered to be excellent and used for further analysis. The radiologist with 10 years of experience was responsible for measuring the remaining IVIM DW image data.

**Figure 1:**
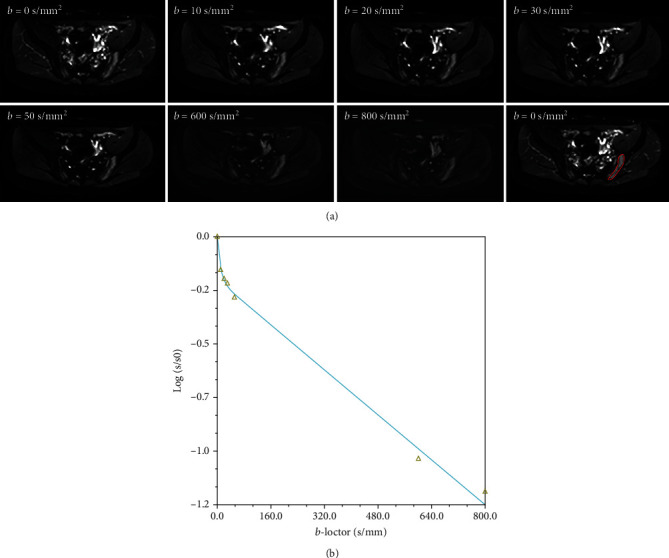
(a) Demonstration of diffusion-weighted images with 7 *b* values from a patient with axial spondyloarthritis (axSpA) and a region of interest (ROI) drawn in the lesion area on DWI of *b* = 0 s/mm^2^. (b) The relationship between signals and *b* values for the lesion in the sacroiliac joint.

For the axSpA patients with visible bone marrow edema (BME) in SIJs on conventional MR images, only one freehand region of interest (ROI) was drawn in the largest lesion of sacroiliitis on the axial map. If the area of BME was not large enough to draw an ROI, those patients will be included in the Non-BME group. For the Non-BME group, ROIs were placed in the subchondral lesions in the sacrum or the ilium. To reduce the impact of ROI on our results, the size of each ROI was chosen to be as large as possible with the exclusion of the blood vessels, adjacent bone cortex, cystic areas, necrosis, and fat metaplasia. For the healthy volunteers, ROIs were placed in the subchondral marrow in the middle areas of the sacrum or the ilium along the SIJs. The mean area of ROI was 24.08 mm^2^ (range 18.5-36.7 mm^2^). And then, the same ROIs were automatically replicated and pasted on the corresponding area on the *D*_fast_ and *f* maps at the same level. At last, the same ROI masks were propagated to all the other *b* values images (Figure 1).

According to the IVIM DWI theory proposed by Le Bihan et al. [7], DWI based on multiple *b* values is implemented in a biexponential model as

(1) $$\begin{array}{c} \frac{{\text{S}}_{\text{b}}}{{\text{S}}_{0}}=\left(1-\text{f}\right)\exp\left(-\text{b Dslow}\right)+\text{f }\exp\left(-\text{b Dfast}\right), \end{array}$$

where *S*_*b*_ represented the signal intensity of the pixel when the diffusion gradient is on and *S*_0_ represents the signal intensity of the pixel when the diffusion gradient is off [20]. *D*_slow_ was obtained by a simplified linear fitting equation with b values higher than 200 s/mm^2^ as Equation (2) [21]:

(2) $$\begin{array}{c} R^{2}=\frac{1‐\text{SSE}}{{\text{SS}}_{\text{total}}} \end{array}$$

was applied to assess the consistency of fit, where SSE indicates the error sum of squares between the fitted curve and data and SS_total_ is defined as the total sum of squares between all the calculated values and their overall average.

To acquire high diagnostic performance, a threshold *b* value with the ability to separate the active groups from inactive groups was defined as the optimal threshold *b* value. Three threshold *b* values encompassing 10, 20, and 30 s/mm^2^ were tested. If threshold *b* value was chosen to be 10 s/mm^2^, then *b* values of 10, 20, 30, 50, 600, and 800 s/mm^2^ were used to measure *D*_slow_. With threshold *b* value of 30 s/mm^2^, then, *D*_slow_ was obtained using *b* values including 30, 50, 600, and 800 s/mm^2^.

### 2.4. Statistical Analysis

Statistical analysis was performed with Statistical Product and Service Solutions (SPSS) version 22.0 (IBM, Armonk, NY). All measurements were expressed as the mean ± standard deviation (SD) and were illustrated with Bland–Altman plot. The examinee and patient were grouped as follows: active group (*n* = 40) vs. the inactive group (*n* = 144), active group vs. the control group (*n* = 20), and inactive group vs. the control group. The Kruskal-Wallis test was used to test the difference among the three groups. The parameters (*D*_slow_, *f*, and *D*_fast_) between every two groups were separately tested by the one-way ANOVA test. Receiver operating characteristic (ROC) curve analysis was used to evaluate the diagnostic performance of *D*_slow_, *f*, and *D*_fast_ between two groups. Diagnostic performance of area under the curve (AUC) values was defined as follows: <0.7, low; 0.7–0.9, medium; and >0.9, high, respectively. *P* < 0.017 was considered statistically significant.

## 3. Results

### 3.1. Patient and Lesion Characteristics

According to our inclusion and exclusion criteria, a total of 184 patients (137 males, 47 females; mean age, 28.62 ± 9.26 years; age range, 12–64 years) with axSpA were eventually enrolled in this study, including 40 patients (28%) in the active group and 144 patients (72%) in inactive groups. Patient characteristics are shown in Table 2. No statistical differences were identified between the active and inactive groups for age, gender, disease duration, and BASDAI score (all *P* < 0.05), while both ESR and CRP had a significant difference between the two groups. For BME lesions, hypointense or isointense lesions were observed in sacral and iliac bones of the sacroiliac joint on TSE T1W images and *f* maps, and hyperintense lesions in affected areas were shown on axial T2W and SPAIR T2W images and Dsmaps. 34 patients (85%) with BME and 6 patients (15%) with non-BME were in the active group, while 117 patients (81.51%) with non-BME and 27 patients (18.49%) with BME were in the inactive group.

### 3.2. Comparison of Parameters Derived from IVIM DWI in the Three Groups with Different Threshold *b* Values

Excellent intra- and interobserver agreement of *D*_slow_, *f*, and *D*_fast_ could be found in Table 3. For any threshold *b* value studied for the intraobserver correlation coefficient, the lowest mean values of *D*_slow_, *f*, and *D*_fast_ are 0.895, 0.832, and 0.873, respectively. For any threshold *b* value studied for the interobserver correlation coefficient, the lowest values of *D*_slow_, *f*, and *D*_fast_ are 0.993, 0.848 and 0.859, respectively.

**Figure 2:**
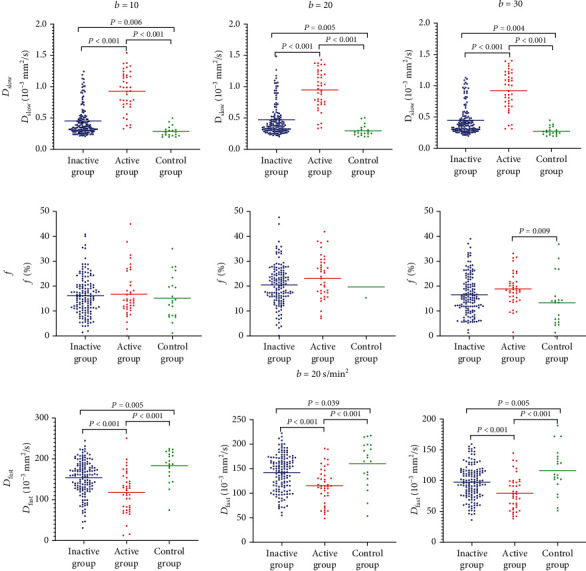
Scattered plots for *D*_slow_, *f*, and *D*_fast_ in the active, inactive, and control groups. All data were analyzed by analysis of variance and tested by one-way ANOVA test with *P* value < 0.05 as a statistically significant difference.

As shown in Figure 1(b), the signal intensity of all *b* values fitted well to the biexponential model with a *R*^2^ = 1‐SSE/SS_total_. (Figure 1(b)). The comparison of *D*_slow_, *D*_fast_, and *f* of the three groups is described in Table 4 and Figure 2 with different threshold *b* values. We found significant differences among the three groups for *D*_slow_ and *D*_fast_ adopting threshold *b* values of 10, 20, or 30 s/mm^2^ (all *P* < 0.001). Moreover, *D*_slow_ and *D*_fast_ were statistically different between any two groups (all *P* < 0.05). In terms of *f*, significant differences were observed in the active group vs. the inactive group and the active group vs. control group utilizing optimal threshold *b* value of 30 s/mm^2^ (both *P* < 0.05), while there was no difference between every two remaining groups with threshold *b* values of 10, 20, and 30 s/mm^2^ (all *P* ≥ 0.05).

### 3.3. ROC Curve Analysis for Parameters Derived from IVIM DWI for the Detection of the Activity in axSpA Patients

**Figure 3:**
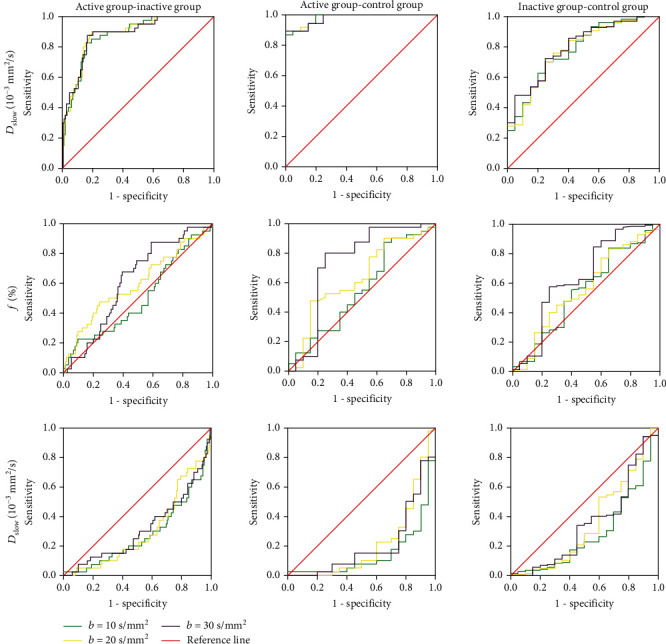
The receiver operating characteristic (ROC) curve for *D*_slow_, *f*, and *D*_fast_ in the active, inactive, and control groups (threshold *b* value: 30 s/mm^2^).

Table 5 and Figure 3 show the three-group ROC analysis of IVIM parameters using threshold *b* values of 10, 20, and 30 s/mm^2^. *D*_slow_ demonstrated the most perfect differentiation between the active and the control groups with high AUC values with threshold *b* values of 10, 20, and 30 s/mm^2^ (0.981, 0.981, and 0.980, respectively). And it achieved accuracy of 0.900, 0.933, and 0.933 with sensitivity of 0.875, 0.900, and 0.900 and specificity of 1.000, 1.000, and 1.000, respectively. Also, it provided moderate performance to discriminate active groups from inactive groups and inactive groups from control groups with medium AUC values with threshold *b* values of 10, 20, and 30 s/mm^2^. *f* only showed the moderate performance to separate the active group from the control groups with the medium AUC value of 0.738 and the cutoff value of 14.486% utilizing the optimal threshold *b* value of 30 s/mm^2^. With the increasing of the threshold *b* value (from 10 to 30 s/mm^2^), the AUC among three groups also increased. *D*_fast_ presented low performance (all AUC < 0.35) with threshold *b* values of 10 and 30.

## 4. Discussion

Our study investigated the performance of *D*_slow_, *f*, and *D*_fast_ to detect the activity in patients with axSpA using IVIM DWI with threshold *b* values of 10, 20, and 30 s/mm^2^. We confirmed that the optimal threshold *b* value was 30 s/mm^2^. Using the optimal threshold *b* value of 30 s/mm^2^, *D*_slow_ provided the best performance to detect the activity in axSpA patients, and *f* showed the moderate performance to discriminate the axSpA patients in the active stage from those in the inactive stage, while the disease activity could not be diagnosed by *D*_fast_ in axSpA patients.

In our study, a large sample size of 184 patients was analyzed, and thus, the results based on the optimal threshold *b* values were more persuasive for the detection of the axSpA activity in sacroiliitis. Previous studies confirmed that, with a carefully selected threshold *b* value, the error could be reduced in the measurement of parameters from IVIM DWI [22, 23]. However, it is demonstrated that the optimal threshold *b* value is dependent on the location of the body, such as 40 s/mm^2^ in breast cancer and 100 s/mm^2^ in cervical adenocarcinoma [24, 25]. In this study, we found the optimal threshold for sacroiliitis was 30 s/mm^2^ in axSpA patients, which has not seen reported. Compared with the threshold *b* values of 10 and 20 s/mm^2^, a b value of 30 s/mm^2^ increases the AUC (cutoff value) for perfusion parameters (*f* and *D*_fast_) between the active and the inactive groups (the result is provided in Table 5 and also shown in Figure 3). Previous studies showed that *f* and *D*_fast_ had no statistical differences between the active and inactive groups in axSpA patients, so perfusion parameters derived from IVIM DWI could not be used to distinguish the active from the inactive axSpA patients [10]. However, our results confirmed that the activity could be detected by *f* in axSpA patients and the AUC for *f* was 0.613 (13.743) between the active and the inactive groups with the threshold *b* values of 30 s/mm^2^. This improvement was expected to be potential clinical values for detecting the activity of the axSpA patients.

Furthermore, which is due to the different effect on *D*_slow_ from the threshold *b* values of 10, 20, and 30 s/mm^2^. Therefore, the results for *D*_slow_ presented in Figure 2 should be identical. The authors should explain why there are differences. *b* value = 200 s/mm^2^ has been popularly used to detect the disease activity of axSpA patients for the IVIM DWI threshold [8, 12]. When using a threshold *b* value of 200 s/mm^2^, the contributions of the perfusion or diffusion to *D*_slow_ or *f*/D_fast_ are considered to be neglect. In our study, the results of *D*_slow_ presented differences in the same way for the three different cases; however, there is no statistical difference between them. We considered that the threshold *b* values of 10, 20, and 30 s/mm^2^ effected on diffusion to *D*_slow_. Recently, several studies suggested that this threshold *b* value may be too high as the turning point of a biexponential model is generally around 50 s/mm^2^ [15]. Our results also suggested a *b* value of 30 s/mm^2^ should be selected for separating active patients with axSpA from inactive ones, which also corresponds to the turning point of the biexponential model for the IVIM DWI fitting curve.

So far as we know, it remains unclear whether *f* can identify the axSpA activity using the full IVIM model [12]. In this study, we disclosed that *f* may have the ability to detect the axSpA activity with the optimal threshold b value of 30 s/mm^2^. Active sacroiliitis increases the ratio of extracellular water to intracellular water and the movement of water molecules, which is also associated with increased microvascular perfusion[26]. In previous studies, *D*_slow_ has been confirmed to be the most reliable parameter among the three parameters (*D*_slow_, *f*, and D_fast_) for detecting the activity of sacroiliitis in axSpA patients [8, 10, 11, 27]. Although the *b* value of 0 s/mm^2^ was excluded in our measurements, *D*_slow_ with threshold *b* values of 10, 20, and 30 s/mm^2^ was still the most effective indicator of disease activity. *f* and *D*_fast_ in the IVIM model is related to perfusion [28]. The optimal threshold *b* value dramatically improved the diagnostic performance for *f*. *D*_fast_ demonstrated the poor diagnostic performance despite using any threshold *b* value, in agreement with the result reported for the full IVIM model by Li et al. [15]. This result can be explained that *D*_fast_ tends to be unstable unless an unrealistically high signal-to-noise ratio (SNR) is achieved to an SNR of >122.48, while *f* can reach to be steady at a moderate SNR of 40.48 [29]. Thereby, a combination of *D*_slow_ and *f* with the optimal threshold value of 30 s/mm^2^ could supply more accurate information to detect the axSpA activity.

Several limitations remained in current our study. Firstly, taking scanning times for patients into account, the relatively fewer numbers of 7 *b* values were utilized to probe threshold *b* values in this study, which might reduce the accuracy of the parameters from IVIM DWI. Generally, the optimal *b* value distribution could dramatically amplify IVIM parameter reliability, and the number of threshold *b* values ranging from 6 to 12 is even suggested to be the minimal applied number in liver disease detection [30, 31]. Second, the relationship between bone mineral density (BMD) and IVIM parameters is not covered in our study. Some studies believe that IVIM parameters are affected by BMD[32, 33]. Hence, an investigation of the influence of BMD on IVIM DWI is desirable in sacroiliitis in the future. Thirdly, *b* values that be selected and evaluated for the biexponential algorithm in this study are quite arbitrary and empirical, which violates the free distribution of parameters. In future studies, more *b* values will be studied and evaluated.

In conclusion, this study confirmed that *D*_slow_ has been confirmed to be the most reliable parameter among the three parameters (*D*_slow_, *f*, and *D*_fast_) for detecting the activity of sacroiliitis in axSpA patients. *f* will promisingly increase the diagnostic accuracy of the axSpA activity to improve treatment for patients with axSpA using the optimal threshold *b* value of 30 s/mm^2^, whereas *D*_fast_ will not.

## Figures and Tables

**Table 1:** Summary of MRI scan parameters.

Sequences	TSE T1WI mDixon	TSE T2WI mDixon	T1-TSE	T2W-SPAIR	IVIM DWI
Plane	Coronal	Coronal	Axial	Axial	Axial
TR/TE (ms)	569/20	2300/85	500/10	5200/70	4000/63.5
Thickness (mm)	3	3	6	5	4
Field of view (mm)	230 × 332	230 × 319	332 × 353	300 × 400	300 × 360
Intersection gap (mm)	0.5	0.5	1	0.5	1
SENSE factor	2.6	2	1.8	2.3	2.8
Matrix	272 × 325	288 × 394	272 × 343	236 × 406	100 × 120
Number of signal average (NSA)	1	1.5	1	1.2	2

IVIM DWI was based on *b* values = 0, 10, 20, 30, 50, 600, and 800 s/mm^2^.

**Table 2:** Summary of axSpA patients' characteristics.

Parameter	Active group	Inactive group	*P* value
Age (y)	29.95 ± 9.92	28.25 ± 9.03	0.31
Gender (male/female)^∗^	31/9	106/38	0.62
Disease duration (m)	54.90 ± 62.42	45.37 ± 46.69	0.37
ESR (mm/h)	29.24 ± 24.58	10.27 ± 9.81	<0.001
CRP (mg/dL)	24.27 ± 40.18	6.48 ± 12.59	0.01
BASDAI score	3.47 ± 2.26	2.53 ± 1.72	0.08

Except where indicated, data are the means ± standard deviations. AxSpA: axial spondyloarthritis; BME: bone marrow edema; ESR: erythrocyte sedimentation rate; CRP: C-reactive protein; BASDAI: bath ankylosing spondylitis disease activity index. ^∗^Data are numbers of participants.

**Table 3:** Intra- and interobserver agreement in the assessment of *D*_slow_, *f*, and *D*_fast_.

Parameters	Intra- and interclass coefficient correlation (95% CI)	
	Intraobserver	Interobserver
Threshold *b* = 10 s/mm^2^		
*D*_slow_ (×10^−3^ mm^2^/s)	0.992 (0.985-0.996)	0.993 (0.987-0.997)
*f* (%)	0.859 (0.750-0.923)	0.954 (0.915-0.975)
*D*_fast_ (×10^−3^ mm^2^/s)	0.873 (0.775-0.931)	0.914 (0.841-0.954)
Threshold *b* = 20 s/mm^2^		
*D*_slow_ (×10^−3^ mm^2^/s)	0.990 (0.980-0.995)	0.994 (0.988-0.997)
*f* (%)	0.923 (0.859-0.958)	0.848 (0.721-0.919)
*D*_fast_ (×10^−3^ mm^2^/s)	0.894 (0.809-0.942)	0.949 (0.907-0.973)
Threshold *b* = 30 s/mm^2^		
*D*_slow_ (×10^−3^ mm^2^/s)	0.895 (0.811-0.943)	0.995 (0.991-0.997)
*f* (%)	0.832 (0.705-0.907)	0.899 (0.818-0.945)
*D*_fast_ (×10^−3^ mm^2^/s)	0.897 (0.814-0.944)	0.898 (0.816-0.945)

*D*_slow_: pure diffusion coefficient; *f*: perfusion fraction; *D*_fast_: pseudoperfusion coefficient; CI: confidence interval.

**Table 4:** Comparison of *D*_slow_, *f*, and *D*_fast_ of patients in the active, inactive, and control groups using different threshold *b* values.

Parameter	Active group	Inactive group	Control group	*P*
Threshold *b* = 10 s/mm^2^				
*D*_slow_ (10^−3^ mm^2^/s)	0.953 ± 0.300	0.405 ± 0.171^#^	0.286 ± 0.080^#,^^∗^	<0.001
*f* (%)	17.953 ± 9.251	15.068 ± 6.674	15.091 ± 8.396	0.751
*D*_fast_ (10^−3^ mm^2^/s)	108.971 ± 45.037	153.790 ± 42.069^#^	182.938 ± 37.665^#,^^∗^	<0.001
Threshold *b* = 20 s/mm^2^				
*D*_slow_ (10^−3^ mm^2^/s)	0.980 ± 0.284	0.414 ± 0.174^#^	0.293 ± 0.085^#,^^∗^	<0.001
*f* (%)	24.105 ± 8.481	20.416 ± 7.033	19.673 ± 9.562	0.172
*D*_fast_ (10^−3^ mm^2^/s)	110.590 ± 35.003	141.334 ± 34.207^#^	159.995 ± 45.047^#,^^∗^	<0.001
Threshold *b* = 30 s/mm^2^				
*D*_slow_ (10^−3^ mm^2^/s)	0.973 ± 0.286	0.408 ± 0.171^#^	0.283 ± 0.069^#,^^∗^	<0.001
*f* (%)	19.985 ± 5.757	16.934 ± 7.437	13.293 ± 9.853^#^	0.031
*D*_fast_ (10^−3^ mm^2^/s)	79.748 ± 28.815	98.595 ± 26.569^#^	116.155 ± 36.301^#,^^∗^	<0.001

Except where indicated, data are the means ± standard deviations. *D*_slow_: pure diffusion coefficient; *f*: perfusion fraction; *D*_fast_: pseudoperfusion coefficient. ^#^*P* < 0.05 vs. the active group; ^∗^*P* < 0.05 vs. the inactive group. *P* < 0.017 was considered statistically significant.

**Table 5:** ROC curve analysis for *D*_slow_, *f*, and *D*_fast_ in the active, inactive, and control groups.

Parameter	Active group-inactive group AUC [cutoff value] (sensitivity, specificity, accuracy)	Active group-control group AUC [cutoff value] (sensitivity, specificity, accuracy)	Inactive group-control group AUC [cutoff value] (sensitivity, specificity, accuracy)
Threshold *b* = 10 s/mm^2^			
*D*_slow_ (10^−3^ mm^2^/s)	0.877 [0.654]	0.981 [0.501]	0.778 [0.307]
	(0.825, 0.840, 0.837)	(0.875, >0.999, 0.900)	(0.701, 0.750, 0.707)
*f* (%)	0.499 [-] (-)	0.551 [8.510] (0.875, 0.350, 0.700)	0.548 [8.614] (0.840, 0.350, 0.780)
*D*_fast_ (10^−3^ mm^2^/s)	0.283 [-] (-)	0.145 [-] (-)	0.283 [-] (-)
Threshold *b* = 20 s/mm^2^			
*D*_slow_ (10^−3^ mm^2^/s)	0.882 [0.578]	0.981 [0.538]	0.776 [0.298]
	(0.900, 0.806, 0.821)	(0.900, >0.999, 0.933)	(0.757, 0.700, 0.756)
*f* (%)	0.595 [25.343]	0.632 [25.164]	0.565 [13.669]
	(0.475, 0.757, 0.696)	(0.475, 0.850, 0.600)	(0.833, 0.350, 0.774)
*D*_fast_ (10^−3^ mm^2^/s)	0.306 [-] (-)	0.214 [-] (-)	0.352 [-] (-)
Threshold *b* = 30 s/mm^2^			
*D*_slow_ (10^−3^ mm^2^/s)	0.881 [0.605]	0.980 [0.519]	0.794 [0.304]
	(0.875, 0.833, 0.842)	(0.900, >0.999, 0.933)	(0.722, 0.750, 0.720)
*f* (%)	0.613 [13.743]	0.738 [14.486]	0.644 [14.474]
	(0.875, 0.410, 0.512)	(0.800, 0.750, 0.783)	(0.576, 0.750, 0.598)
*D*_fast_ (10^−3^ mm^2^/s)	0.316 [-] (-)	0.205 [-] (-)	0.346 [-] (-)

Data are areas under the curve. Numbers in parentheses are cutoff values. AUC: area under the curve; ROC: receiver operating characteristic; *D*_slow_: pure diffusion coefficient; *f*: perfusion fraction; *D*_fast_: pseudoperfusion coefficient.

## Data Availability

All the data related to this study are mentioned in the manuscript. Any further data if required may be obtained on request from the corresponding author.

## Abbreviations

IVIM: Intravoxel incoherent motion
DWI: Diffusion-weighted imaging
axSpA: Axial spondyloarthritis
AUC: Area under the curve
SIJ: Sacroiliac joints
MRI: Magnetic resonance imaging
AxSpA: Axial spondyloarthritis
SNR: Signal-to-noise ratio
IRB: Institutional review board
ESR: Erythrocyte sedimentation rate
CRP: C-reactive protein
ASDAS: Spondyloarthritis disease activity score
ROI: Region of interest
ICC: Intraclass correlation coefficient
BME: Bone marrow edema
SSE: Sum of squares due to error
SPSS: Statistical product and service solutions
IBM: International business machines
SD: Standard deviation
ROC: Receiver operating characteristic
BMD: Bone mineral density.
